# Genetic Risk Profiling in Parkinson’s Disease and Utilizing Genetics to Gain Insight into Disease-Related Biological Pathways

**DOI:** 10.3390/ijms21197332

**Published:** 2020-10-04

**Authors:** Ashley Hall, Sara Bandres-Ciga, Monica Diez-Fairen, John P. Quinn, Kimberley J. Billingsley

**Affiliations:** 1 Department of Pharmacology and Therapeutics, Institute of Systems, Molecular & Integrative Biology, University of Liverpool, L69 7BE, UK; ashley.hall@liverpool.ac.uk (A.H.); jquinn@liverpool.ac.uk (J.P.Q.); 2 Molecular Genetics Section, Laboratory of Neurogenetics, National Institute on Aging, National Institutes of Health, Bethesda, MD 20892, USA; sara.bandresciga@nih.gov; 3 Neurogenetics Group, University Hospital MutuaTerrassa, Sant Antoni 19, 08221 Terrassa, Barcelona, Spain; monicadifa@gmail.com

**Keywords:** Parkinson’s disease, genetic risk, biological pathways

## Abstract

Parkinson’s disease (PD) is a complex disorder underpinned by both environmental and genetic factors. The latter only began to be understood around two decades ago, but since then great inroads have rapidly been made into deconvoluting the genetic component of PD. In particular, recent large-scale projects such as genome-wide association (GWA) studies have provided insight into the genetic risk factors associated with genetically ‘’complex’’ PD (PD that cannot readily be attributed to single deleterious mutations). Here, we discuss the plethora of genetic information provided by PD GWA studies and how this may be utilized to generate polygenic risk scores (PRS), which may be used in the prediction of risk and trajectory of PD. We also comment on how pathway-specific genetic profiling can be used to gain insight into PD-related biological pathways, and how this may be further utilized to nominate causal PD genes and potentially druggable therapeutic targets. Finally, we outline the current limits of our understanding of PD genetics and the potential contribution of variation currently uncaptured in genetic studies, focusing here on uncatalogued structural variants.

## 1. Parkinson’s Disease Genetics

Parkinson’s disease (PD) is characterized by a loss of dopaminergic neurons, particularly in the substantia nigra, and widespread accumulation of intracellular α-synuclein protein aggregates such as Lewy bodies. Symptoms include motor features such as bradykinesia (slowness of movement), rigidity, resting tremor and loss of balance, amongst others [1]. A majority of PD patients also have non-motor symptoms, including REM (rapid eye movement) sleep perturbation, cognitive impairment, mood disorders, constipation, sensory symptoms such as hyposmia (reduced sense of smell), and chronic pain [2]. PD is a heterogeneous disorder in which these symptoms may manifest in a spectrum of varying combinations, highlighting that clinical presentation and progression is likely influenced by a mixture of genetic and environmental factors.

The global prevalence of PD is expected to double from 6.2 million cases in 2015 to 12.9 million by 2040 [3]. PD therefore represents a growing challenge for society, since current treatments only manage symptoms. It is imperative that a better understanding of the etiology of PD is achieved to lay the groundwork for treatments that may halt or reverse its progression.

Until only two decades ago, PD was considered to be wholly caused by environmental factors. Early epidemiology studies pointed to exposure to viruses and neurotoxins such as MPTP [4]; perhaps most famous was the strong association between the 1918 influenza pandemic and the increased rates of post-encephalitic parkinsonism that followed. This non-genetic basis of PD was supported by the first cross-sectional twin studies of the disease [5]. It is now known, however, that PD is a complex disorder influenced by both genetic and environmental factors. Indeed, 5–10% of PD follows a classical Mendelian inheritance pattern, and around 15% of PD patients have family history of PD [6]. The first direct evidence of a heritable component of PD came in 1997 with the identification of rare mutations in SNCA (encoding α-synuclein) that were responsible for a form of monogenic PD [7]. This was shortly followed by the discovery of additional rare recessive forms of PD caused by deleterious mutations in PRKN [8], PINK1 (also known as PARK6) [9] and DJ-1 [10], and the identification of autosomal dominant PD resulting from mutations in LRRK2 [11]. To date, mutations within several additional genes have been associated with monogenic PD and have been discussed previously [12].

The majority of PD cases cannot be attributed to a single penetrant, deleterious mutation. Considering this, many studies have pursued the ‘’common disease common variant’’ (CDCV) hypothesis, in which the genetic component of PD is considered to be the cumulative result of many common, low-risk alleles [13]. Genome-wide association (GWA) studies have been instrumental in addressing this hypothesis. Briefly, the premise of a GWA study is to compare the genotypes of many individuals, often in a case vs. control setup, and determine which common genetic variants, typically single nucleotide variants (SNVs), are consistently associated with a trait. The power of this analysis increases with the number of participants, as this allows the contributions of relatively low-risk disease-associated variants to be detected. Accordingly, PD GWA studies of increasing size have been performed across several populations [14,15,16,17,18,19]. The most recent and largest PD GWA study meta-analysis identified 90 independent genetic signals associated with genetically complex PD and involved approximately 37,700 cases, 18,600 ‘proxy’ cases and 1.4 million controls [20]. However, it has proven difficult to directly ascribe function to these disease-associated SNVs because most occur in non-coding DNA. Any regulatory effects upon genes may, therefore, be subtle and difficult to study, and SNVs may not necessarily exert their effects on the nearest gene. Furthermore, a genetic signal may simply be in linkage disequilibrium (LD) with the true disease-causing variant. To help researchers prioritize candidate genes associated with GWA study signals, a Parkinson’s disease GWA study locus browser application has been recently developed that compiles functional data, genetic data and literature reviews for PD-associated loci [21] (https://pdgenetics.shinyapps.io/GWASBrowser/).

Here, we comment on how this wealth of genomic information can be utilized to build genetic risk scores, which can be useful in the prediction of disease risk and progression. Further, we note how large-scale pathway-specific genetic risk profiling can give insight into disease-related biological pathways. Utilizing this data and leveraging new analytical approaches can further identify specific genes potentially linked to PD. Finally, despite the huge success over the last decade in identifying PD genetic risk factors, we outline the current gaps in our understanding of PD genetics, with a focus on the contribution of the uncharacterized parts of the human genome, namely, uncatalogued structural variants.

## 2. Genetic risk Profiling in Parkinson’s Disease

**Figure 1:**
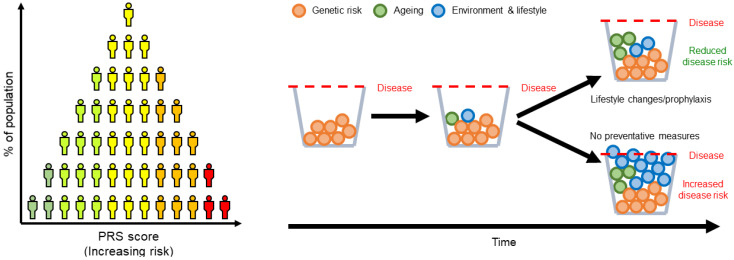
Polygenic risk scores based on the weighted sum of genetic variants can be used to estimate an individual’s predisposition to complex polygenic disorders such as Parkinson’s disease (PD), and allow those at greatest risk in the population to be identified (left). Those at risk can be recommended lifestyle changes or preventative treatments that may combat their increased likelihood of developing a disease (right).

Each risk allele identified by GWA studies confers relatively little risk when considered individually. Polygenic risk scores (PRS) are simple models that sum the contribution of multiple risk variants of variable effect sizes, as determined by GWA studies summary statistics (Figure 1). In this way, the information representing an individual’s genetic architecture is aggregated to form a picture of their total known genetic risk for disease. PRS can therefore be potentially used to identify at-risk individuals and allow implementation of early lifestyle changes or prophylaxis, although most commonly PRS is used in concert with other disease predictors. As the sample sizes involved in GWA studies have grown, PRS have been used to analyze the genetic risk of a number of complex disorders such as schizophrenia [22], Alzheimer’s disease [23] and PD [20].

PRS have shown potential as a tool for prediction, with scores having been associated with PD risk [24], age at onset (AAO) [24,25], and increased cognitive and motor decline [26]. The 90 risk loci identified in the largest, most recent PD meta-analysis are associated with higher relative risk of developing PD, with those in the top 10% of PRS being nearly six-fold more likely to develop PD than those in the bottom 10% [20]. It has also been demonstrated that by factoring in other variables such as age, sex, family history and hyposmia, a combined risk score can be produced with considerable sensitivity and specificity [27]. However, at present PRS are not without limitations. PRS may incorporate genetic variants that are not perfectly correlated with the causal factor, leading to uncertainty of variant effect sizes. This reduces the applicability of PRS across populations besides the training population, even if they actually share large effect-size causal variants [28,29]. It is also imperative that a PRS and any associated uncertainty is effectively interpreted, as it may unduly confer stress, a false sense of security, or the wrong decision regarding preventative measures.

It is important to note that several loci associated with PD risk variants are pleomorphic, capable of harboring both rare, deleterious mutations and common variants that confer risk. For example, point mutations and multiplications of SNCA are causative of early-onset autosomal-dominant PD [30,31], while variation in non-coding regions of the locus have been repeatedly associated with increased risk of genetically complex PD [14,18,19]. Additionally, recent GWA studies for PD AAO suggest that not all PD risk alleles influence AAO [32], indicating that overall PD risk and PD AAO may be driven by processes that only partially overlap. Similarly, GWA studies for PD progression (i.e., identifying variants that are associated with how PD changes over time) have identified variants at SLC44A1 and ADRA2A, two loci not currently associated with PD risk, with increased disease progression and development of insomnia, respectively [33]. Taken together, these observations highlight the heterogeneous and multifaceted nature of PD and reinforce the importance of refining PRS; more accurate disease modelling will allow the various molecular and pathological subtypes of PD to be delineated, and disease trajectories to be predicted. In this way, certain PRS may potentially serve as inclusion criteria for clinical trials, thereby enabling more subtype-targeted therapies to be developed. For example, a recent study observed that those with lower PD PRS were at greater risk of developing PD if they were diabetic, but conversely diabetes may have had a protective effect among those with higher PRS for PD [34]. If this stems from the effects of anti-diabetic drugs being modified by genetic risk of PD, then individuals who may benefit from their use could be identified on the basis of their PRS, potentially resulting in more targeted clinical trials.

## 3. Utilizing Genetic Risk Profiling to Identify Biological Pathways Involved in Disease

As noted, there has been great success at identifying genetic risk factors for PD and using this information to build genetic risk scores as a tool for predicting disease risk, AAO and increased cognitive and motor decline [26]. However, there are still major gaps in our understanding of the underlying molecular and cellular processes that are involved in PD. In light of this, recent studies have started to address the latter by constructing pathway-specific genetic risk scores to gain insight into the potential disease-related biological pathways.

First, building on previous work that suggested that the endocytic membrane trafficking pathway (EMTP) could be a driving force in PD pathogenesis [35], Bandres-Ciga et al. ran a systematic analysis to identify the contribution of genetic variants within genes associated with the EMTP to risk of PD. An extensive list of genes that are involved in the EMTP was constructed using the Molecular Signatures Database (MSigDB) of an annotated gene set for “endocytosis” based on the Kyoto Encyclopedia of Genes and Genomes information (KEGG-endocytosis) and a detailed literature search. SNVs within the 262 EMTP-associated genes were extracted from 41,321 individuals (18,869 cases and 22,452 controls) of European ancestry, and an EMTP-specific PRS was built reflecting the cumulative risk of common variants in this pathway. The EMTP risk profiling versus disease status was found to be significantly associated with PD (random-effects *p*-value = 2.55 × 10^−12^, beta = 0.227, SE (standard error) = 0.032) with an odds ratio of 1.20 per standard deviation increase in the PRS from the population mean, confirming a cumulative pathogenic effect of EMPT variants in PD [36].

Another pathway-specific risk profiling study focused on the role of the mitochondrial function pathway in PD. Although mitochondrial dysfunction has been strongly implicated in the etiology of monogenic forms of PD, the role that mitochondrial processes played in the risk of genetically complex disease was less clear. Two gene lists were curated to encompass different levels of evidence for the involvement of the respective protein products in disease phenotypes that relate to mitochondrial function. The “primary” gene list consisted of genes mutated in mitochondrial disorders (*n* = 196). The “secondary” gene list was a broader list (*n* = 1487), curated using the OMIM API to identify all genes for which the word “mitochondria” (or derivatives) appeared in the free-text description, and this was combined with MitoCarta v2.0 genes with no OMIM phenotype. A mitochondrial function pathway-specific PRS was constructed for both lists, again from 41,321 individuals (18,869 cases and 22,452 controls) of European ancestry. Even when the known PD risk loci [20] were removed from the primary gene list, the pathway was still significantly associated with PD with an odds ratio of 1.12 per standard deviation increase in the PRS from the population mean (random-effects *p*-value = 6.00 × 10^−4^, beta = 0.11, SE = 0.03). The secondary gene list was also associated with PD with an odds ratio of 1.28 per standard deviation increase in the PRS from the population mean (random-effects *p*-value = 1.9 × 10^−22^, beta = 0.25, SE = 0.03). Further, the mitochondrial function pathway-specific PRS was identified to be significantly associated with later AAO of PD [37]. Overall, this study provided robust evidence that mitochondrial processes are drivers of genetically complex PD.

**Figure 2:**
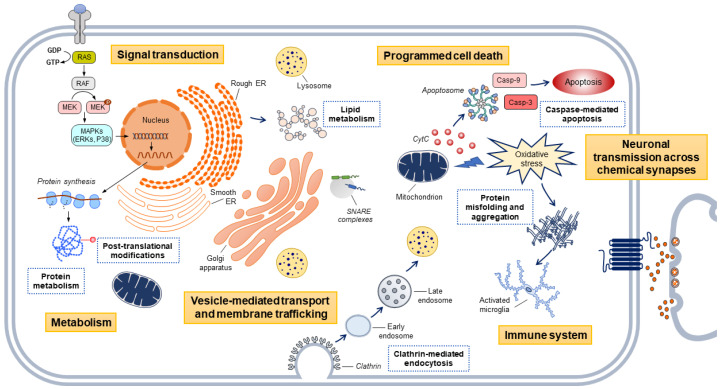
Main biological pathways associated with PD risk through genetic risk profiling. Yellow squares indicate the hierarchies in which the pathways are clustered according to published literature and public curated databases. Blue squares indicate the pathways that contribute the most to PD etiology. Key: CytC, Cytochrome C; ER, endoplasmic reticulum.

Most significantly, a recent large-scale analysis implemented a high-throughput and hypothesis-free approach to assess the role of PD risk in over two thousand curated and well-defined gene sets. The analysis was split into a discovery and replication phase. The discovery dataset consisted of 7218 PD cases and 9424 controls, and the replication dataset, which was used to validate the results, consisted of 5429 PD cases and 5814 controls. Summary statistics from Chang et al. 2017 [19] were used as the reference dataset to perform the analysis. The MSigDB was used to extract gene sets representative of biological pathways, and a pathway-specific polygenic effect score was constructed for each dataset. In support of the previous pathway-specific analyses [36,37], the study found a significant association between PD risk and the endocytic membrane trafficking and mitochondrial function pathways [38]. Most notably, this approach identified significant associations between risk of PD and novel biological pathways. Overall, the study concluded that the main contributors to PD etiology are molecular processes underlying protein misfolding and aggregation, post-translational protein modification, immune response, membrane and intracellular trafficking, lipid metabolism, synaptic transmission, endosomal–lysosomal dysfunction and apoptosis mediated by initiator and executioner caspases (Figure 2). This was the first study to test the contribution of thousands of molecular processes to PD risk in an unbiased and data-driven manner and provide a foundational resource for the PD community through a publicly available pathways browser [38] (https://pdgenetics.shinyapps.io/pathwaysbrowser/).

In addition to relating PD-related SNVs to functional pathways, a key step in unravelling PD etiology is to determine in which cell types these variants are biologically relevant. The genes highlighted in the most recent PD GWAS meta-analysis were found to enriched for expression exclusively in brain-derived tissues, including the substantia nigra, frontal cortex and cerebellum [20]. Recent single-nuclei transcriptomics in the substantia nigra support association of PD with dopaminergic neuron-specific gene expression and, interestingly, implicate oligodendrocyte-specific expression [39]. Similar single-cell transcriptomics in the mouse nervous system also confirm the association between dopaminergic neurons and PD, and reiterate the novel association with oligodendrocytes [40]. Additionally, the pathway analysis study mentioned previously integrated single-cell expression data to link PD risk to expression patterns in dopaminergic neurons, serotonergic neurons, hypothalamic GABAergic neurons and neural progenitors [38].

A caveat to these pathway approaches, and indeed much research utilizing GWAS signals, is that it is assumed that SNVs in noncoding regions affect the nearest gene, although it is increasingly acknowledged that they can regulate genes over 10 kb away [41,42]. Recently, H-MAGMA was developed from MAGMA, a tool for linking GWAS risk signals to cognate genes, by incorporating HiC chromatin structure information in order to map SNVs to distal genes they interact with via chromatin looping [43]. This approach also revealed that these genes underwent developmental stage-specific and cell type-specific interactions in several neurobiological disorders, demonstrating how greater functional understanding of GWAS signals can be achieved when they are considered in their cellular context. Another recent study identified PD-relevant pathways via analysis of co-expressed modules of genes, the expression of which was perturbed in brain regions susceptible to PD [44].

## 4. From Pathway to Possible Therapeutic Target: Analytical Methods for Gene Prioritization

Although GWA studies have been a key approach to identify loci linked to PD etiology, it is widely assumed that the top genetic variants underlying such associations are not usually the disease-causing variants. GWA studies signals are often non-coding and lie on intergenic or regulatory regions of the genome, making interpretation complex. The mapping resolution of a GWA study is limited by the LD structure of the genome [45], and therefore understanding what the true causal variants are and how they disrupt molecular mechanisms contributing to disease remains challenging.

As previously mentioned, pathway-specific genetic risk profiling is one approach to identify general biological processes involved in disease. Expanding on this, it is possible to further leverage this data and newly developed statistical tooling to then nominate specific genes in the associated pathways that are related to PD risk. Several integrative analytic approaches have been developed in an attempt to prioritize functional genes or regulatory elements that may reveal druggable targets amenable to therapeutic intervention. Summary-data-based Mendelian randomization (SMR) arose as a promising methodology that allows the use of GWA studies and expression or methylation quantitative trait loci (eQTL and mQTL) data from independent studies to explore the possibility of GWA study variants affecting phenotype through genetic regulation of a transcriptional output (changes either in expression or methylation at CpG sites) [46]. The premises are that if the expression or methylation of a gene is influenced by a genetic variant (QTL), then there will be differences in gene expression or methylation levels among individuals carrying different genotypes of the genetic variant. Perhaps one of the strengths of this methodology is that it has the ability to distinguish whether changes in gene expression or methylation and the phenotype of interest are associated owing to a single shared genetic variant (pleiotropic model), or in contrast, whether there are two or more distant genetic variants in LD affecting gene expression and the phenotype of interest independently (linkage model).

In the PD genetics arena, SMR has been widely applied to prioritize genes from GWA studies signals. A curious example is illustrated in the last PD GWA study meta-analysis [20] in which a novel multi-signal locus (UBTF-GRN-FAM171A2) comprising independent genome-wide associated SNVs was identified. SMR analyses showed that GRN, encoding progranulin, is the most putative causal gene within this locus, and that disease risk might be explained by modulation of progranulin expression in this instance. GRN has previously been associated with frontotemporal dementia [47], and mutations within this gene have been shown to be connected with lysosomal storage disorders and neuronal ceroid lipofuscinosis [48], making this target an interesting PD candidate to further study.

Furthermore, SMR has also been successfully used to explore enrichment of eQTLs or mQTLs linked to certain biological processes involved in PD, such as the mitochondrial pathway [37] and the EMTP [36]. Among the most recent state-of-the-art functional genomics methods implemented in the PD genetics field, the use of colocalization and fine-mapping should be highlighted. Unlike SMR, this type of analysis consists of a Bayesian framework that uses summary data (usually from eQTL and GWA studies) to estimate signals that colocalize with expression changes by applying posterior probabilities [49]. In PD, a recent colocalization study of publicly available brain eQTL and PD GWA study summary statistics reinforced the RAB7L1 gene as the priority candidate for the chromosome 1q32 locus PD risk association [50]. In fact, additional studies have provided further functional evidence linking RAB7L1 to LRRK2 [51] by implicating RAB7L1 as a substrate for LRRK2 kinase activity [52,53,54].

Lastly, transcriptome-wide association studies (TWAS) have been powerful strategies to integrate large-scale functional genomics data with GWA studies to characterize the functional effects of associated variants [55]. TWAS integrates SNV-expression correlation (cis-SNV effect sizes), GWA study summary statistics and LD reference panels to impute RNA expression levels onto large cohorts of individuals to identify putative genes involved in disease. In the PD genetics field, TWAS has been applied to dissect the effect of genetic variation on RNA expression and splicing in order to prioritize disease-relevant genes. As an example, a recent PD TWAS has prioritized potential targets whose predicted expression or splicing levels in peripheral monocytes cells and in dorsolateral prefrontal cortex are significantly associated with PD risk, advancing our understanding of PD in a genomic context [56]. Additionally, dysregulation of small noncoding transcripts, particularly microRNAs, is increasingly recognized as a potential mediator of reactive oxygen species production and mitochondrial dysfunction in PD and other neurodegenerative diseases [57]. Recent RNA-seq experiments in longitudinal PD cohorts associated PD with dysregulation of miRNAs implicated in function of mitochondria and inflammatory immune cells such as leukocytes [58].

Despite these advanced methodological approaches, the number of PD loci that are functionally validated remains very low and mostly includes genes that are known to cause monogenic forms of PD. A limitation to SMR, TWAS and fine-mapping/colocalization methods is that they operate under different assumptions. Additionally, it is likely that the genes prioritized with one methodology and using data from a particular tissue do not replicate when using a different approach in another tissue.

## 5. The Limitations in Our Current Understanding of Parkinson’s Disease Genetics, Focusing on the Contribution of Structural Variants

By providing insight into disease-related biological processes, the ultimate aim of genetic research is to support the development of therapeutic treatments that stop or slow disease. Gaining a full understanding of the genetic factors that contribute to PD is essential for achieving the latter. As previously noted, the field has seen great success in identifying genetic variants that influence PD risk, onset, and progression. Despite these efforts, our understanding of the genetic factors that contribute to PD is incomplete. The largest PD GWA study to date identified that when leveraging current GWA study datasets and genetic risk profiling methods, only around 16–36% of the heritable component of PD can be explained. This large gap in our understanding of PD genetics is often referred to as the “missing heritability”. Many factors likely constitute the “missing heritability”, including uncaptured genetic variation represented by (1) rare variants, (2) epigenetics and (3) structural variants (SV). As the contributions of both (1) and (2) have been explored in great detail in recent reviews [59,60], here we focus instead on the contribution of (3) SVs.

Unlike SNVs (a change in only one nucleotide), SV are somewhat arbitrarily defined as DNA rearrangements that involve at least fifty nucleotides. They can be divided into subclasses that consist of unbalanced copy number variants (CNVs), which include deletions, duplications and insertions, of novel sequence, as well as balanced rearrangements, such as interchromosomal and intrachromosomal translocations and inversions. SVs also include transposable element (TE) insertions, segmental duplications, multi-allelic CNVs of highly variable copy number and complex rearrangements that consist of multiple combinations of these described events. SV can have a huge phenotypic impact by either disrupting gene function and regulation or modifying gene dosage. Further, numerous studies have highlighted the role of SV in functional changes across human populations and cell and tissue types [61,62,63]. Detection of SV from short-read sequencing data is problematic, as the evidence for SV resembles common sequencing and alignment artefacts. However, following the recent rapid development of improved sequencing and bioinformatic tooling that can more accurately call SV, it is now apparent that human genomes differ more as a consequence of SV than as a result of a point mutation [64,65,66,67,68]. For comparison, based on SNVs alone, on average the genomic variation between two individuals is 0.1%; however, when SV are incorporated, this increases over ten-fold, to 1.5% [69]. SV therefore represent the majority of the genetic variation in the human genome, yet this form of genetic variation remains mainly uncatalogued.

In regards to the role of SV in PD, our current understanding of the genetic basis of genetically complex PD has been predominantly gained through GWA studies that utilize genome-wide SNV-chip based datasets. Hence SV represent a substantial and important mutational force that has not yet been systematically addressed. On the other hand, our understanding of the genetic basis of monogenic forms of PD has been primarily obtained using more targeted approaches, such as linkage analysis and sequencing of genes in candidate intervals. As a result of these analyses, it has been repeatedly identified that SV are causal variants of many forms of monogenic PD and Parkinsonism, with examples including causative CNVs, repeat expansions and TE insertions.

CNV SV causative of monogenic forms of PD have been discussed in great detail in recent reviews [70,71]. In brief, the first major discovery to highlight the role of SV in PD was in 2003, whereby Singleton et al. identified a causative triplication (CNV) of the entire genomic region that encompassed the gene *SNCA* in a large family with autosomal dominant PD [31]. Multiplications of *SNCA* were later reported in several other families from different ancestral backgrounds, including other *SNCA* triplications [72,73,74,75,76,77,78,79,80] and, more commonly, duplications of the gene [81,82,83,84,85,86,87]. Overall, the severity of the clinical phenotype of *SNCA* multiplications is associated with gene dosage and mRNA/protein expression levels in brain [73]. Hence, patients with *SNCA* triplication have rapidly progressive symptoms (generally in the fourth decade) whereas phenotypically *SNCA* duplication patients usually resemble late-onset genetically complex PD cases [81,82]. Further, causative CNVs have been reported in other familiar PD genes, such as the genes *PARK2* [8], * PINK1* [88] and *DJ-1* [10].

Other classes of SV have been associated with forms of PD and Parkinsonism. One prominent example is the TE SINE-VNTR-Alu (SVA) insertion in intron 32 of the Transcription initiation factor TFIID subunit 1 (*TAF1*) gene, which is causative of X-Linked Dystonia Parkinsonism (XDP). The XDP TE SVA insertion is not only variable in its presence/absence in the genome but between individuals with the XDP TE SVA, it is also variable in the size of one of the SVAs repeat domain. The variation in the XDP TE SVAs CT elements is disease-modifying, as the size of the SVAs hexanucleotide CT repeat domain inversely correlates with XDP age at onset [89]. The XDP TE SVA insertion was previously found to alter sequence within *TAF1* introns causing abnormal mRNA expression and significant dysregulation of a neural-specific TAF1 isoform—N-TAF1—in XDP causative relative to control brain tissue [90]. In addition, generated XDP and matched control induced pluripotent stem cell (iPSC) lines confirmed TAF1 transcript dysregulation and also revealed a significant decrease in expression of TAF1 transcript fragments that span the region of the SVA (intron 32–36). Remarkably CRISPR/Cas9 excision of the SVA rescued the aberrant transcriptional signature and normalized expression of TAF1 in patient-derived iPSCs [91].

Despite the evidence that SV are causative of many forms of monogenic PD and Parkinsonism, SV are yet to be directly assessed in a large-scale and whole-genome manner in genetically complex PD cases. However, one recent example that highlights the importance of characterizing a structural variant with such an approach identified that deletions at 22q11.2 are associated with genetically complex PD. Deletions at 22q11.2 were previously associated with a wide range of clinical syndromes, including 22q11.2 deletion syndrome, Schizophrenia [92] and rare reported cases of Parkinsonism and PD [93,94,95]. Consequently, Mok et al. screened data from independent GWA studies to systematically establish the frequency of 22q11.2 deletions in ~9k PD cases and ~14k controls. This large-scale analysis identified that deletions at 22q11.2 increase the risk of PD, particularly early-onset PD (onset age <45 years) [96]. Overall, few other complex trait GWA studies have directly assessed SV, although rare and de novo SV have been implicated in the genetics of other complex genetic diseases such as autism [97,98,99], and schizophrenia [100,101,102,103]. The current lack of large-scale SV studies for complex genetic diseases is mainly due to two reasons. The first is that previous tooling has not been able to accurately call SV at scale. The second is that there has been a lack of a comprehensive SV reference set. Fortunately, several groups are pioneering large-scale efforts to further develop structural variant detection tooling and to establish population-scale structural variant reference sets for the broader research community. One of the largest SV studies to date detected and genotyped SV in 14,891 genomes from diverse populations. Following this, Collins et al. discovered over 400,000 novel SV and identified that SV are responsible for 25–29% of all rare protein-truncating events per genome. Further, to provide an SV reference map, these data were made publicly available through the publicly available gnomAD browser (https://gnomad.broadinstitute.org/downloads/) [104].

In sum, as we enter an era of whole-genome and long-read sequencing, it is evident that to gain a complete understanding of the genetics of PD, SV will need to be routinely incorporated into GWA studies. The current state of SV calling has been compared to SNV calling a decade ago [105]. Therefore, in the context of PD it is important to reflect on how the great success of the last decade in PD genetics was made with the SNV based studies, i.e., through the use of well-powered unbiased GWA studies. Hence, moving forward, to systematically address SV in PD it will be crucial to leverage the newly developed accurate SV callers and perform large-scale SV oriented genetic studies.

## 6. Future Directions

Over the last decade, tremendous advances have been made in PD genetics, improving our ability to understand and define the cumulative risk of disease. The new era holds promise, and success will only be possible through working collaboratively and openly sharing data, processes and results. In this context, the Global Parkinson’s Genetics Program (GP2) (https://parkinsonsroadmap.org/roadmap/) will further our understanding of the genetic architecture of PD by first expanding the sample size of GWA studies to include over 150,000 individuals from all over the world. This will have a special focus on under-represented populations for fine-mapping and trans-ethnic admixture analysis. Secondly, GP2 will facilitate the application of bioinformatic methodologies by promoting open science and connecting researchers worldwide. Finally, GP2 will help unmask the contribution of currently uncaptured genetic variation, such as SV and rare variation, to the risk of PD.

## Abbreviations

PD	Parkinson’s Disease
REM	Rapid eye movement
GWA	Genome-wide association
PRS	Polygenic risk score
CDCV	Common disease common variant
SNV	Single nucleotide variants
LD	Linkage disequilibrium
AAO	Age at onset
EMTP	Endocytic membrane trafficking pathway
MSigDB	Molecular Signatures Database
KEGG	Kyoto Encyclopedia of Genes and Genomes
SE	Standard error
SMR	Summary-data-based Mendelian randomization
QTL	Quantitative trait loci
TWAS	Transcriptome-wide association studies
SV	Structural variants
CNV	Copy number variant
GP2	Global Parkinson’s Genetics Program
